# Förder- und Rahmenbedingungen für Partizipative Gesundheitsforschung aus Projektsicht

**DOI:** 10.1007/s00103-020-03274-w

**Published:** 2021-01-05

**Authors:** Andreas Bethmann, Birgit Behrisch, Sebastian von Peter

**Affiliations:** 1 Katholische Hochschule für Sozialwesen Berlin, Köpenicker Allee 39–57, 10318 Berlin, Deutschland; 2 Medizinische Hochschule Brandenburg Theodor Fontane, Neuruppin, Deutschland

**Keywords:** Teilhabe, Gesundheitsförderung, Forschungsförderung, Antragstellung, Co-Forschende, Participation, Health Promotion, Research funding, Proposal writing, Co-researchers

## Abstract

Partizipative Gesundheitsforschung ist ein eigenständiger Forschungsansatz, der eigene Förderbedingungen benötigt. In der Zeitschrift *Gesundheitswesen* erschien im April 2020 ein Artikel, der Empfehlungen für geeignete Maßnahmen zur Förderung von partizipativer Gesundheitsforschung diskutiert. Im Rahmen des vorliegenden Beitrags werden dort angesprochenen Aspekte durch Praxisbeispiele untermauert.

3 Erfahrungsbeispiele werden aufgeführt, um die vielfältigen und unterschiedlichen Bedingungen während der Bewerbung um Mittel für partizipative Forschungsprojekte zu erläutern. Beispiel 1 zeigt, wie die fruchtbare Zusammenarbeit von Antragstellenden und fördernder Stelle partizipative Prozesse ermöglichte. In Beispiel 2 haben die Förder- und Antragsbedingungen, trotz eines Fokus auf Partizipation, eine wirksame Beteiligung im Rahmen der Antragsentwicklung nicht möglich gemacht. Beispiel 3 setzt sich mit eigenen, berechtigten Forschungsinteressen von Betroffenen auseinander, die in der gegenwärtigen Fördererlandschaft nur eingeschränkt Widerhall finden und kaum Förderung erfahren.

Die Erfahrungen zur Förderung partizipativ angelegter Forschung gestalten sich sehr unterschiedlich. Es gibt positive Fälle, es überwiegen aber Erfahrungen mit Hindernissen, die eine partizipative Zusammenarbeit erschweren. Speziell betrifft dies die gemeinsame Erarbeitung von Forschungsanträgen (Themen, Fragestellung, Design) durch alle maßgeblichen Akteur*innen. Um partizipative Gesundheitsforschung effektiv zu fördern, braucht es daher flexiblere Ausschreibungsformate mit längeren Antragsfristen, die eine mehrstufige Förderung erlauben und auch für nichtakademische Akteur*innen und Akteursgruppen zugänglich sind.

## Abstract (en)

Participatory Health Research is a unique research approach that needs unique funding conditions. In April 2020, the German journal *Gesundheitswesen* published an article that recommends reasonable measures for funding Participatory Health Research. Some of the aspects mentioned are illustrated in the present article with practical examples.

Three examples of experiences are introduced to explain the manifold and different conditions for applying for funds for participatory research projects. Example 1 shows good cooperation between the applicant and funder, allowing participatory processes. The conditions for funding in example 2 – despite being focused on participatory involvement – did not allow effective participation. Example 3 deals with the personal, legitimate research interests of affected people, who are only partially reflected in the current funding landscape and hardly receive any funding.

The experiences regarding the funding of participatory research are quite different. There are positive cases, however experiences in which applicants meet obstacles that hinder participative collaboration prevail. This particularly concerns the joint development of research tenders (issues, research questions, design) by all relevant stakeholders. Therefore, to effectively foster participatory health research, more calls for proposals are needed that are flexible, have prolonged application periods, allow multilevel funding, and are open to nonacademic stakeholders.

## Einleitung

Die partnerschaftliche Forschung von Wissenschaftler*innen gemeinsam mit verschiedenen Co-Forschenden aus Lebenswelt, Berufspraxis, Administration und Politik hat eine lange Tradition und wird in vielen Ländern und in unterschiedlichen Konzeptionen vertreten. In den Gesundheitswissenschaften etabliert sich diesbezüglich der Ansatz der Partizipativen Gesundheitsforschung (PGF; [1])/Participatory Health Research (PHR; [2]). PGF vertritt den Anspruch, unterschiedliche Perspektiven aller Akteur*innen für und in der Forschung sichtbar zu machen und Wissens- und Ergebnisbestände mittels Koproduktion zu erarbeiten. Aufgrund der derzeitigen hierarchischen Ordnung der verschiedenen Wissenstypen von Wissenschaft, Praxis, Lebenswelt o. Ä. wird dabei auch immer deren Machtgefüge und -gefälle zueinander thematisiert. Diese Art zu forschen zeigt in der Anwendung des Ansatzes Konsequenzen in allen Phasen eines Forschungsprozesses und unterscheidet sich von konventionellen Ansätzen der Gesundheitsforschung. Daher brauchen partizipative Forschungsprojekte andere Organisationsformen und nicht zuletzt angepasste finanzielle Förderstrukturen, um ihrem Anspruch von Partizipation in der Wissensgenerierung gerecht werden zu können.

Der vorliegende Text bezieht sich auf den Artikel „Partizipative Gesundheitsforschung in Deutschland – quo vadis?“ („Quo vadis?“), der der Frage nachgeht, wie sich PGF in Deutschland in geeigneter Weise fördern lässt. „Quo vadis?“ basiert auf Erfahrungen, Erkenntnissen und Analysen aus dem *Netzwerk Partizipative Gesundheitsforschung* (PartNet^1^; [3]) und stellt Ansätze zur sinnvollen Fortentwicklung der Förderung von PGF vor. So sollten partizipative Prozesse strukturell gefördert werden, indem etwa die Mitarbeit von Bürger*innen und Patient*innen angemessen vergütet und zusätzliche Aufwände von partizipativen Prozessen in der Forschung finanziell gefördert werden. Partizipation sollte als konkret zu realisierendes Kriterium in Förderprogrammen und Ausschreibungen verankert werden, wie dies zum Teil auch schon erfolgt. Dies beinhaltet die Aufforderung, in Anträgen zu Partizipation als Kriterium Stellung zu nehmen (ohne jedoch verpflichtend zu sein!), flexible Ausschreibungsformate zu schaffen, in denen partizipative Projekte gezielt gefördert werden, Gutachter*innen bei der Bewertung von Partizipation zu unterstützen sowie formative Evaluationen von partizipativen Projekten und generell eine partizipative Methodenentwicklung sicherzustellen. Zudem sollte die Vernetzung von partizipativ Forschenden gefördert werden.

Der vorliegende Artikel ist eine Ergänzung zu „Quo vadis?“. Er macht in der Vorstellung verschiedener Projekte Erfahrungen sichtbar, die zu den o. a. Schlussfolgerungen geführt haben. Ein Beispiel für eine gelungene Förderung partizipativer Forschung durch einen gemeinsamen Lernprozess zwischen Antragstellenden und dem Projektträger ist der Forschungsverbund *PartKommPlus*. Daran anschließend beschreiben Erfahrungen mit der Förderlinie „Klinische Studien mit hoher Relevanz für die Patientenversorgung“, wie Förderbedingungen Partizipation behindern können, obwohl diese gleichzeitig gefordert ist. Wie schwierig es für Selbstvertretungsorganisationen der Zivilgesellschaft ist, öffentliche Förderung für ihre Forschungsinteressen und -zielsetzungen zu erhalten, verdeutlicht das Projekt „Alt werden von Menschen mit Kleinwuchs“. Alle Beispiele illustrieren, wie Förderbedingungen im Wortsinn förderlich, aber eben auch ein großes Hemmnis für die Durchführung partizipativ angelegter Forschung sein können. Auf diese Weise werden verschiedene Perspektiven unterschiedlicher Akteur*innen in einen gemeinsamen Dialog gesetzt, der sich als Beitrag zur Diskussion um die Weiterentwicklung der Förderung von PGF versteht.

## Förderung partizipativer Forschung im Zusammenwirken von Geförderten und Fördernden: der Forschungsverbund PartKommPlus

Im Hinblick auf partizipative Forschung und deren Förderung ist die Entstehungsgeschichte des Forschungsverbundes PartKommPlus [4] bemerkenswert. Die „Richtlinien zur Förderung von Forschungsverbünden zur Primärprävention und Gesundheitsförderung“ vom 12.06.2013 des Bundesministeriums für Bildung und Forschung (BMBF) [5] sahen nicht direkt partizipative Ansätze vor, boten aber viel Raum für die Möglichkeit, solche Forschungsansätze einzubeziehen. Formulierungen wie „zielgruppenspezifisch“ oder „Transfer in die Praxis“ waren Anker, um ausdrücklich partizipative Forschungsansätze wie auch die Beforschung partizipativer Methoden ins Zentrum der Arbeit des zukünftigen Forschungsverbundes zu rücken. Mehr noch galt dies für die förderungswürdigen Themenschwerpunkte (z. B. Prävention und Gesundheitsförderung bei spezifischen Zielgruppen, vor allem bei vulnerablen Bevölkerungsgruppen) und Forschungsansätze (z. B. Erforschung der Faktoren, die die Umsetzung von Präventionsmaßnahmen unter Alltagsbedingungen beeinflussen).

Die Projekte in PartKommPlus wurden von PartNet-Mitgliedern erarbeitet, die sich im Vorfeld schon längere Zeit mit PGF beschäftigt hatten. Dabei waren zum Teil bereits Netzwerke vorhanden, die einen leichten Zugang zu den später mitforschenden Akteur*innen ermöglichten, zum anderen wurden die ersten Verbindungen im Zuge des Antragsprozesses aufgenommen. Um diese Zeit wusste man aus internationalen Projekten bereits, dass idealerweise schon die Forschungsfragen mit allen Akteursgruppen gemeinsam erarbeitet werden sollten [6, 7]. Doch war es im Umfeld der deutschen Förderlandschaft und bei einer Abgabefrist von lediglich 6 Monaten unmöglich, dies umzusetzen. Infolgedessen waren es dort, wo nicht bereits Netzwerke zu später mitforschenden Akteur*innen aus Adressat*innengruppen und aus der Praxis vorhanden waren, überwiegend die Forschenden, die die Forschungsfragen und das Design der jeweiligen Projekte bestimmten.

In der ersten Stufe des zweistufigen Antragsprozesses wurden die in englischer Sprache verfassten Anträge durch ein internationales Gutachtergremium beurteilt, das in seinen Bewertungen ausdrücklich auf den Aspekt der Partizipation einging und diesen positiv aufnahm. Ob aus diesem oder einem anderen Grund, in der zweiten Phase der Antragstellung gab es einen relativ intensiven Austausch zwischen dem Projektträger und den antragstellenden Projekten sowie der Verbundkoordination hinsichtlich der Ausrichtung des Budgets auf partizipative Prozesse. Projektträger (und Ministerium) zeigten sich hier sehr flexibel und bereit, auf die speziellen Bedarfe partizipativer Forschung einzugehen, soweit die Richtlinien dies zuließen. Den Antragstellenden verlangte dies ab, präzise Begründungen zu liefern, was zu einem geschärften Bewusstsein für die speziellen Belange partizipativer Forschung führte.

So wurden z. B. Einrichtungen außerhalb der Wissenschaft direkt gefördert, Forschungsprojekte (mit-)zuleiten, Peer-Forschende und Praxispartner*innen bekamen Aufwandsentschädigungen. Besonders hervorzuheben ist die Finanzierung von Dolmetschdiensten, ein sehr ungewöhnlicher Vorgang bei Förderungen durch das BMBF. Dies geschah vor dem Hintergrund, dass im Antrag bereits der Anschluss an den internationalen Diskurs vorgesehen war, der durch die Begleitung durch Expert*innen der International Collaboration for Participatory Health Research (ICPHR) gesichert werden sollte. Dolmetscher*innen sollten den reibungslosen Dialog zwischen den internationalen Expert*innen und Partnern aus Praxis und Adressat*innengruppen ermöglichen. Mit der Finanzierung wurde anerkannt, dass die Mitforschenden nicht nur aus wissenschaftlichen Einrichtungen kamen und daher Englisch als Arbeitssprache nicht selbstverständlich war. Damit wurden auch die partizipativ angelegten Arbeitsprozesse von PartKommPlus gewürdigt.

Hier wie an anderen Stellen erwies sich, dass Geldgeber*innen (und deren exekutive Vertreter) ein wesentlicher Teil des partizipativen Forschungsprozesses sind. Im Fall von PartKommPlus standen sie dabei außerhalb des eigentlichen Prozesses, begleiteten diesen aber unterstützend im Rahmen der durch Gesetze und Richtlinien vorgegebenen Möglichkeiten, zuweilen auch beratend. Auf diese Weise wurde es erst möglich, dass z. B. die Prozesse zur Vertrauensbildung und in der Folge auch der Anpassung der Forschungsfragen durch gemeinsame Diskurse von professionell Forschenden, Adressat*innen, Partner*innen aus der Praxis und weiteren Akteur*innen genügend Zeit und Raum bekommen konnten.

In diesem Fall scheint es, dass es einen gemeinsamen Lernprozess von Zuwendungsempfänger und Zuwendungsgeber gegeben hat, der für die Ausgestaltung der Förderung von PGF-Projekten von großem Wert ist.

## Partizipation in der klinischen Forschung: Fallstricke von Ausschreibungs- und Designvorgaben

Die folgenden Ausführungen basieren auf einer (versucht) partizipativen Antragstellung durch Mitglieder der „AG Psychiatrische Versorgungsforschung der Medizinischen Hochschule Brandenburg“ (AG)^2^ im Jahr 2018 auf die Ausschreibung des BMBF „Klinische Studien mit hoher Relevanz für die Patientenversorgung“ [8]. Im Vergleich zum Vorjahr hatte das Ministerium im Jahr 2018 das Kriterium der Partizipation deutlich stärker in der Ausschreibung verankert, sowohl auf der Ebene der Zielstellung als auch auf der Ebene der Begutachtungskriterien. Dort hieß es beispielsweise, dass eine Förderung nur dann gewährt werde, wenn die „Bedürfnisse der Patientinnen und Patienten angemessen berücksichtigt werden“; die „Relevanz der Fragestellung für Patientinnen und Patienten“ müsse gesichert und „Patientinnen und Patienten, bzw. deren Vertretungen in der Planung und Durchführung des Projektes“ beteiligt werden. Diese Entwicklungen waren umso erfreulicher, als es diese BMBF-Ausschreibungslinie zwar seit vielen Jahren gibt, das Kriterium der Partizipation aber noch im Vorjahr keine Rolle gespielt hatte [9]. In der Umsetzung der Antragsformulierung stieß man dennoch auf einige, teils auch grundsätzliche Probleme.

Das erste Problem für die AG war die kurze Antragsfrist: Vom Zeitpunkt der Ausschreibung am 09.02.2018 bis zur Einreichungsfrist am 28.05.2018 blieben nur 3,5 Monate Zeit, um interessierte Akteur*innen und/oder Akteursgruppen anzusprechen und mit ihnen am Antrag zu arbeiten. Das ist ein Zeitraum, der für jede Form der partizipativen Zusammenarbeit eher kurz ist, selbst wenn man im Vorfeld schon die relevanten Akteur*innen und Akteursgruppen und mit ihnen zusammen die relevanten Fragen identifiziert hat. Partizipation braucht Zeit, will sie wirksam sein und sich von vornherein an den Anliegen und Perspektiven derjenigen orientieren, um die es in der Fragestellung zentral geht. Zum Glück gab es in der AG auch vor der Antragstellung schon einige Verbindungen zu Akteur*innen der Selbsthilfe und -vertretung, sodass eine Co-Forschenden-Gruppe relativ bald zusammengestellt war. Dennoch führte die knapp bemessene Antragsfrist dazu, dass sich diese Akteur*innen in kürzester Zeit in den Stand der Literatur einlesen und mit teils sehr komplexen Vorstudien beschäftigen mussten, um überhaupt eigene Entscheidungen treffen zu können.

Zweitens sah die BMBF-Ausschreibung zum damaligen Zeitpunkt keine Beantragung einer Vorphase vor, was bedeutet, dass für die partizipative Zusammenarbeit während der Antragstellung keine Mittel abgerechnet werden konnten. Viele der üblicherweise beteiligten Akteur*innen und Akteursgruppen werden in fast allen Kontexten dazu aufgerufen, sich ausschließlich nur ehrenamtlich einzubringen, in Gremien, in der Selbstvertretung, in der Forschung. Dies widerspricht der Forderung, z. B. seitens PartNet, dass für wirksame Beteiligung finanzielle Entlohnung unerlässlich ist [3]. Will man eine Symmetrie in der Zusammenarbeit schaffen, braucht es Mittel, um entstandene Aufwände zu entschädigen und eingebrachte Expertise zu entlohnen. Da sich die AG Psychiatrische Versorgungsforschung damals noch im Aufbau befand, konnten für die Antragsentwicklung keine Aufwandsentschädigungen angeboten werden, sodass sich die Gruppe der Co-Forschenden ehrenamtlich beteiligt hat. Motivierend in diesem Zusammenhang war, dass sich die zu beforschende Intervention in ihrer Ausrichtung weitgehend mit den Interessen der Gruppe deckte.

Drittens wurden nur „Hochschulen und außeruniversitäre Forschungseinrichtungen sowie Einrichtungen und Träger der Gesundheitsversorgung“ als antragsberechtigt genannt. Ein partizipativer Bottom-up-Antrag, beispielsweise aus der Selbsthilfe oder -vertretung heraus, war demnach prinzipiell ausgeschlossen, was faktisch bedeutete, dass interessierte Akteur*innen auf die Ansprache und das Wohlwollen von Forschungsinstitutionen angewiesen waren. Im Zusammenhang der Antragsentwicklung hat die AG lange darüber gesprochen, welche Effekte eine „Einladung“ von Co-Forschenden durch eine wissenschaftliche Institution auf die darauffolgende Zusammenarbeit hat. Unter anderem steckt diese von Anfang an Ziele fest und lässt dadurch weniger Spielräume für partizipatives Aushandeln.

Schließlich, und das ist am wichtigsten, macht das BMBF für die Antragslinie „Klinische Forschung“ sehr strenge Designvorgaben. Klinische Studien wurden in der Ausschreibung von 2018 als „multizentrische, prospektive, kontrollierte Studien zum Wirksamkeitsnachweis von Therapiekonzepten“ definiert. Es ging um die Evaluation umschriebener Interventionen und vor allem eine „konfirmatorische Zielsetzung“. Eine solche Definition von klinischen Studien entspricht fachwissenschaftlichen Standards, lässt aber nur wenig Raum für partizipative Aushandlungsprozesse und gemeinsam getroffene Entscheidungen. In diesem Fall hat bspw. die geforderte konfirmatorische Zielsetzung der beantragten Studie den Bezug auf Vorstudien notwendig gemacht, was wiederum die Möglichkeiten einer partizipativen Aushandlung für oder gegen ein primäres Outcome-Maß stark eingeschränkt hat. Außerdem ließ das eher normierte methodische Raster einer kontrollierten Studie wenig Spielräume für davon abweichende methodische Vorgehensweisen.

Trotz dieser Herausforderungen wurde am Ende der Antrag eingereicht, ist aber leider nicht bewilligt worden. Die Zusammenarbeit war dennoch lehrreich, v. a. in Hinblick auf die Frage von günstigen Rahmenbedingungen für eine partizipative Antragsstellung. Die hauptsächliche Erkenntnis der AG war, dass zu enge Designvorgaben die Gefahr bergen, immer ähnliches Wissen hervorzubringen [10]. Wenn Partizipation als Kriterium in der Forschungsförderung genutzt wird, sollte also kritisch überprüft werden, an welcher Stelle das erfolgt, in Bezug auf welche Art der Forschung und auf welche Fragestellungen.

## Bottom-up-Forschung: Wenn Betroffene berechtigte Interessen haben

Die vorangegangenen Beispiele schildern die Vorgehensweise partizipativer Umsetzung im Rahmen bestehender Förderausschreibungen und -programme. Das Projekt „Alt werden von Menschen mit Kleinwuchs“, ein Kooperationsprojekt des *BundesselbsthilfeVerbands Kleinwüchsiger Menschen e.* *V.* (VKM) und des Instituts Mensch, Ethik und Wissenschaft (IMEW), wurde in der Themenwahl von einer Organisation der Selbstvertretung behinderter Menschen selbst initiiert und stand damit vor den anders gelagerten Schwierigkeiten, sowohl wissenschaftliches Engagement als auch finanzielle Fördermöglichkeiten für die eigenen Interessen akquirieren zu müssen. Im Themenfeld Behinderung und psychische Gesundheit werden spätestens seit den 1990er-Jahren Ansätze emanzipatorischer oder betroffenenkontrollierter Forschung diskutiert, die fordern, dass die maßgebliche Kontrolle, Mitwirkung und Entscheidungsmacht im Forschungsprozess bei Menschen mit Eigenerfahrung von Behinderung oder psychischer Erkrankung liegen sollten. Dabei werden die materiellen und sozialen Bedingungen in Forschungsprozessen kritisiert, die eine derartige Forschungsform bislang oftmals verhindern oder erschweren [11–13].

Der VKM beobachtete bei seinen älteren Mitgliedern (dies meint Menschen ab 40/50 Jahren) eine rückläufige Teilnahme an Veranstaltungen sowie ein vorzeitiges Beenden des Arbeits- und Berufslebens. Die Mitglieder des ehrenamtlich arbeitenden Bundesvorstandes vermuteten aufgrund der gesundheitlichen Situation eine eingeschränkte Teilnahme kleinwüchsiger älterer Menschen am gesellschaftlichen Leben und wandten sich mit dem Wunsch einer wissenschaftlichen Klärung an verschiedene Forschungsinstitutionen. In Kooperation mit dem Institut Mensch, Ethik und Wissenschaft (IMEW) erfolgten über 3 Jahre verschiedene Bemühungen in unterschiedlicher Intensität, Förderung für das von der Selbstvertretung als relevant eingestufte Thema einzuwerben, wobei versucht wurde, die Thematik an verschiedene Diskurse von Alter, Behinderung oder Gesundheit anzubinden. Der VKM trat in den meisten Anträgen als Antragsteller auf und war in alle Prozesse involviert, wodurch auch direkt mit der Enttäuschung bei Ablehnung umgegangen werden musste. In einer der Ablehnungen für eine Förderung im Bereich Alter lautete die Begründung, die Gruppe der Menschen mit Kleinwuchs sei allgemein zahlenmäßig zu gering, um von Forschungsinteresse in der Thematik zu sein, und enthielt den Rat, sich in zukünftigen Anträgen mit weiteren (größeren) Zielgruppen behinderter Menschen zusammenzutun. Dieser wissenschaftsdisziplinarische und förderungslogische Schluss, Ergebnisse auf einer abstrakteren Erkenntnisebene zu generieren, verursachte bei den Initiator*innen der Selbstvertretung hingegen Unverständnis, da sie in ihren alltäglichen Belangen keine Anschlussmöglichkeit, z. B. an die hier vorgeschlagene Zielgruppe sehbeeinträchtigter Menschen, sah.

Über die Jahre erwies es sich als schwierig bis unmöglich, eine Projektfinanzierung für das Anliegen des VKM zu akquirieren, was zu einer Erschöpfung bei allen Beteiligten führte. Das Projekt, das dann letztendlich 2013/2014 im Rahmen weniger Monate durchgeführt wurde, ergab sich aus einer Teilfinanzierung des BKK-Dachverbandes über die Selbsthilfeförderung des SGB V. Die Finanzierungsanforderung verlagerte allerdings die Fragestellung auf den Hauptschwerpunkt Gesundheit hin, sodass nun zu Anforderungen an Prävention und Gesundheitsförderung aus der Perspektive alternder Menschen mit Kleinwuchs geforscht wurde. Allerdings bot sich im finanzierten qualitativen Forschungsdesign die Möglichkeit, Fragen zur Teilhabesituation älterer Menschen im Interviewleitfaden anzuhängen, die dann im Rahmen einer Masterarbeit ausgewertet wurden. Ein wenig überpointiert ausgedrückt: Die Fragestellung, die die VKM zur wissenschaftlichen Klärung drängte, konnte allein über eine wissenschaftliche Qualifikationsarbeit bearbeitet werden [14], während die Suche nach einer Forschungsförderung eine Frage erarbeiten ließ, die von den Betroffenen selbst nicht mit oberster Priorität eingestuft wurde [15]. Auf der Erkenntnisebene und für den VKM selbst führte diese Verschränkung der Forschungsperspektiven allerdings zu einem unerwarteten Mehrgewinn. Menschen mit Kleinwuchs beschreiben sich in ihrem eigenen Selbstbild überwiegend eher als kurz gewachsen und nicht als behindert. Mit Zunahme körperlicher Beschwerden im Alter, entstanden durch Über- und Fehlbelastungen in Anpassung an die Normgröße des Alltags, verändert sich jedoch diese Einstellung. Im Rückblick hätten sich die Befragten mehr Aufklärung über Gesundheitsrisiken im Zusammenhang mit dem Kleinwuchs und entsprechende präventive Maßnahmen, dem körperlichen Kräfteabbau entgegenzuwirken, gewünscht.

Insgesamt beantwortete diese partizipativ-kollaborative Forschung [16] einerseits das ursprüngliche Forschungsinteresse des VKM gegenüber den älteren Mitgliedern und brachte gleichzeitig das Thema möglicher Gesundheitsrisiken, relevant auch für jüngere Mitglieder, in die Verbandsarbeit (z. B. auf dem damaligen Jahrestag) ein. Für alle von den Prozessen und geringen Budgets erschöpften Wissenschaftler*innen und ehrenamtlich im Verband Tätigen brachte es zudem die Erkenntnis, dass sich viele lohnenswerte Fragestellungen in der Selbstvertretung finden lassen, deren Ergebnisse auch direkt den Betroffenen zugutekommen [17], dass es aber bis zur Anerkennung dieser Forschungsform noch ein längerer Weg ist und dieser derzeit vor allem nur mit Zähigkeit und Kreativität zu meistern ist.

## Ausblick

„Quo vadis?“ formuliert Erkenntnisse, die aus verschiedenen Förderungserfahrungen gewonnen wurden. Der Artikel stellt damit einen Diskussionsbeitrag in einem weitaus größeren Diskurs dar, der bislang eher unter der Oberfläche wissenschaftlicher partizipativer Forschung zwischen den Akteur*innen stattfindet, hauptsächlich zwischen denen, die Forschung organisieren (also wissenschaftliche Einrichtungen), und denen, die diese Forschung finanzieren. In den Forderungen zur Verbesserung der Förderung partizipativer Forschung spiegeln sich diese Perspektiven und auch der Stand der derzeitigen Diskussion dahin gehend, dass es bereits eine Art von Dialog zwischen Forschung und Förderung gibt. Dieser Dialog ist nicht einheitlich und auch nicht konfliktfrei. PartKommPlus entstand in seiner jetzigen Form nicht zuletzt durch den gemeinsamen Lernprozess der am Antrag Beteiligten, bei den Forschenden ebenso wie bei den Fördernden bzw. deren Vertreter*innen. Dass dieser Lernprozess weiterhin nötig ist, belegt das Beispiel „Klinische Studien mit hoher Relevanz für die Patientenversorgung“. Hier führten unterschiedliche Sichtweisen und Interessen im Diskurs um die Förderung von PGF dazu, dass eine wirksame Beteiligung im Rahmen dieses Antragsverfahrens nur bedingt umzusetzen, bzw. anzulegen war. Partizipation obligatorisch in Förderkriterien aufzunehmen, ohne zugleich auch entsprechende Antragsbedingungen zu schaffen, behindert sowohl partizipative als auch konventionelle Forschungsansätze.

Dennoch ist eine klare gesellschaftliche Entwicklung sichtbar, die PGF als nützlichen, wenn nicht in manchen Bereichen sogar notwendigen Ansatz begreift, wie sich z. B. an der steigenden Zahl an Publikationen und Schwerpunktheften zu dem Thema ablesen lässt. Dies zeigt sich vermehrt auch in Förder- und Vergaberichtlinien, die partizipative Forschung auf der Grundlage von Erfahrungen aus PGF-Projekten finanzieren und so grundlegende Erkenntnisse der PGF anerkennen. Förderbedingungen entstehen im wissenschaftspolitischen Diskurs und werden in Fördervorgaben konkretisiert. An dieser Stelle sei darauf verwiesen, dass momentan die Idee gemeinsamer Forschung von Wissenschaft und weiteren Akteur*innen (jenseits des Gesundheitsbereichs) überwiegend etwas vereinheitlichend und teilweise vereinnahmend in Deutschland unter dem Begriff von „Citizen Science“ geführt wird, auch dort, wo Ausschreibungen themenoffen und in der Breite sämtlicher Disziplinen an der Förderung von bürgerwissenschaftlichen Vorhaben [18] ansetzen. Problematisch daran ist der Umstand, dass sich unter dem Label Citizen Science überwiegend die naturwissenschaftliche Forschung organisiert [19], die Sozial- und Gesundheitswissenschaften sich hingegen eher unter der Bezeichnung partizipativer Forschung ansprechen lassen. Förderrichtlinien mit einem expliziten oder impliziten Bezug auf Citizen Science führen daher möglicherweise (bzw. mit hoher Wahrscheinlichkeit) zum Ausschluss ganzer Fach- und Forschungsdisziplinen, die sich, auch im internationalen Anschluss, unter anderen Begrifflichkeiten zusammenschließen.

Über die bisherige Diskussion hinaus weist das Beispiel des Projekts des BundesselbsthilfeVerbands Kleinwüchsiger Menschen e. V. Diesbezüglich möchten wir die erste Forderung des Artikels „Quo vadis?“, der sich darauf bezieht, Anreize für Partizipation von Bürger*innen und Patient*innen zu schaffen [3], kritisch kommentieren. Viele partizipative Projekte, die mit feststehenden Forschungsfragen nach Co-Forschenden suchen, berichten von schwierigen Rekrutierungsphasen. Hier zeigt sich aber, dass in zivilgesellschaftlichen Organisationen Interesse an wissenschaftlichen Vorhaben besteht – allerdings eben auch unterlegt mit einer eigenständigen Intention. Disabled Persons Organizations (DPOs) im europäischen Raum verfügen selten über Ressourcen, um selbst Forschung durchzuführen, viele Aktivist*innen besitzen aber Forschungserfahrung [20]. Denn DPOs sehen oft in der Zusammenarbeit mit wissenschaftlichen Institutionen einen Gewinn, allerdings wird in der Umsetzung ein geringer akademischer Zugang zu zivilgesellschaftlichen Fragestellungen beklagt (vgl. auch [21, 22]). Das dritte Beispiel wirft die kritische Frage auf, welche gesellschaftlichen Akteur*innen (allein) berechtigt sind, Forschungsfragen festzulegen und (steuerfinanzierte) Forschung durchzuführen (vgl. [1]). Deutlich zeigt sich das am Umstand, dass sich einfache biomedizinische Designs in Präventionskonzepten gegen komplexere, gesellschaftskritische Designs durchsetzen, was an anderer Stelle schon zugespitzt als das „Darwinsche Gesetz der Präventionspolitik“ bezeichnet wurde [23].

Letztendlich treffen bei einer gemeinsamen Forschung unterschiedlicher Akteur*innen auch verschiedene Erwartungen an und Vorstellungen von Wissenschaft aufeinander; Forscher*innen haben Forschungsziele und Co-Forschende haben Lebensziele [24]. Wie die aufgeführten Beispiele ebenfalls zeigen, sind Verständigungsprozesse zwischen Co-Forschenden und Wissenschaftler*innen für beide Seiten herausfordernd und brauchen Zeit (vgl. auch [25]). Dies liegt auch darin begründet, dass sich Alltags- und Wissenschaftssprache graduell in ihrem Abstraktionsgrad und reflexiven Gehalt unterscheiden und daher politische oder soziale Probleme nicht ohne Weiteres in wissenschaftliche Fragestellungen übersetzt werden können. Trotz dieser Herausforderungen ist umgekehrt zu fragen, ob auf das partizipative Moment, unterschiedliche Perspektiven zusammenzubringen, im entscheidenden Beginn eines Forschungsprozesses wirklich verzichtet werden kann. Co-Forschende verfügen zumeist über „Innensichten“ [26, S. 253] bzw. binden wissenschaftliches, nach Disziplinen geteiltes Wissen als „Wissen aus einer Hand“ zusammen [1, S. 312]. Dadurch können reale Differenzen zwischen Rhetorik und täglicher Lebenserfahrung aufgedeckt werden, die ein essenzielles Antidot gegen politische, administrative und professionelle Routinen darstellen [27].

In ethischer Hinsicht ist zudem zu fragen, inwieweit die ethische Symmetrie [28] verletzt wird, wenn Antragsformulierungen nicht mit den Co-Forschenden abgestimmt werden. Denn Forschungsfragen bestimmen den gesamten Forschungsprozess von Inhalt, Methode und Zielsetzung. Partizipative Prozesse verlangen mit den unterschiedlichen (Macht‑)Asymmetrien in Forschungsprozessen derart reflexiv umzugehen, dass sich für Co-Forschende aus ihrem sozialen Status heraus keine Nachteile ergeben.

Im Sinne dieser Argumentation möchten wir die im Artikel „Quo vadis?“ erhobene Forderung nach flexibleren Ausschreibungsformaten mit längeren Antragsfristen, die eine mehrstufige Förderung erlauben und auch nichtakademischen Akteur*innen und Akteursgruppen zugänglich sind, nachdrücklich unterstützen. Partizipative Forschung beginnt in der Antragsstellung und braucht dafür bereits in der Vorbereitung von Vorhaben einen finanziellen und organisatorischen Rahmen, „damit über Ziele, Design und die Weiterentwicklung eines Projektes partizipativ entschieden werden kann“ [3, S. 330]. Nicht zuletzt ermöglicht eine gehaltvolle und bereits partizipativ angedachte partizipative Ausrichtung des Forschungsvorhabens, den „Diskrepanzen zwischen Antragslyrik und Umsetzung“ [29, S. 162] im Sinne von Scheinpartizipation und Instrumentalisierung entgegenzuwirken.

## Footnotes

1 Weitere Informationen zu PartNet: www.partnet-gesundheit.de.

2 Vgl. https://psychiatrie.immanuel.de/forschung/.
